# Statin-specific inhibition of Rab-GTPase regulates cPKC-mediated IKs internalization

**DOI:** 10.1038/s41598-019-53700-6

**Published:** 2019-11-28

**Authors:** Elsa Ronzier, Xiaorong Xu Parks, Haani Qudsi, Coeli M. Lopes

**Affiliations:** Aab Cardiovascular Research Institute, Department of Medicine, University of Rochester, 601 Elmwood Avenue, Rochester, NY 14642 USA

**Keywords:** Biophysics, Physiology, Cardiology, Molecular medicine

## Abstract

Statins are prescribed for prevention and treatment of coronary artery disease. Statins have different cholesterol lowering abilities, with rosuvastatin and atorvastatin being the most effective, while statins like simvastatin and fluvastatin having lower effectiveness. Statins, in addition to their cholesterol lowering effects, can prevent isoprenylation of Rab-GTPase proteins, a protein family important for the regulation of membrane-bound protein trafficking. Here we show that endosomal localization of Rab-GTPases (Rab5, Rab7 and Rab11) was inhibited in a statin-specific manner, with stronger effects by fluvastatin, followed by simvastatin and atorvastatin, and with a limited effect by rosuvastatin. Fluvastatin inhibition of Rab5 has been shown to mediate cPKC-dependent trafficking regulation of the cardiac delayed rectifier KCNQ1/KCNE1 channels. We observed statin-specific inhibition of channel regulation consistent with statin-specific Rab-GTPase inhibition both in heterologous systems and cardiomyocytes. Our results uncover a non-cholesterol-reducing statin-specific effect of statins. Because Rab-GTPases are important regulators of membrane trafficking they may underlie statin specific pleiotropic effects. Therefore, statin-specificity may allow better treatment tailoring.

## Introduction

Statins (3-hydroxy-3-methylglutaryl-CoA reductase inhibitors) are among the most commonly prescribed drug classes for prevention and treatment of coronary artery disease and their use is expected to increase due to recent changes in therapy guidelines^1^. Statins are known to reduce cardiovascular events and mortality in patients, and studies suggest that statin treatment decrease the incidence of cardiac arrhythmias^2,3^, although the mechanism underlying these effects has not been elucidated. Some studies have investigated the acute effect of statins on cardiac ion channels^4–6^. Our recent work suggested that fluvastatin inhibition of Rab5-prenylation had a protective effect on one of the major repolarizing cardiac channels IKs. We showed that fluvastatin inhibited channel internalization in response to stress stimulus, restoring channel function^7^. Nonetheless, studies on the dose dependence effect of statins and statin specificity of Rab-GTPases are lacking.

Statins have different cholesterol lowering abilities, with rosuvastatin and atorvastatin being the most effective, while statins like simvastatin and fluvastatin are less effective^8,9^. Nonetheless, deciding which statin is the best choice for a specific patient relies not only on its cholesterol lowering ability, but also on other factors. Drug–drug interactions and genetic polymorphisms modulating drug transporter activity are major determinants of different statin pharmacokinetics (for review see^10^). For instance, plasma concentrations of rosuvastatin increased 10-fold during the coadministration of cyclosporine and almost fivefold during the combined administration of lopinavir–ritonavir due to the inhibition of transporter activities^11,12^. Thus, patient’s genetic background, decreased renal function and other concomitant drug treatment have a strong effect on tailoring statin treatment to patients due to drug bioavailability. In addition, statins side effects such as muscle pain, increase in blood sugar levels, liver damage and neurological effects can also guide statin treatment^8,13–15^. Statins also have a number of beneficial off-target effects, which include reduction of the rate of ventricular fibrillation in heart disease patients^2,3^. More recently, in smaller studies, statin therapy was shown to shorten QTc and QTc dispersion in heart failure patients^16^ and suppress superventricular arrhythmias^17,18^. Nonetheless, little is known about the molecular mechanism underlying both beneficial and harmful off-target effects of statins. Without this knowledge, the use of statins for its non-cholesterol-lowering effects is limited.

Statins can be differentiated as either hydrophilic or lipophilic regarding their water solubility. Rosuvastatin, for instance, is hydrophilic. Other statins, such as fluvastatin, simvastatin and atorvastatin have different degrees of hydrophobicity^19–21^. These properties may be important in explaining some of the off-target effects of statins^20,21^. The IKs channel is formed by KCNQ1 and KCNE1 subunits and is one of the major channels responsible for cardiac repolarization. Decrease in channel activity caused by mutations in either subunit is associated with prolongation of QT in the ECG and increased susceptibility for cardiac arrhythmias and sudden death^22^. Our recent study suggested that fluvastatin regulation of the IKs channel may have a protective effect of preventing IKs reduction in response to prolonged stress stimulus^7^. However, the effect of other statins on IKs membrane expression has not been studied. Here we hypothesize that because statins may have different abilities to regulate intracellular membrane endosomes due to their hydrophobicity, statin regulation of Rab-GTPase is statin-specific, and that statin-specificity can be used to target Rab-mediated ion channel regulation.

Small GTPases of the Rab family are key regulators of membrane trafficking and membrane targeting^23,24^. Over 60 members of this family have been identified in humans with specialized roles^25^. For instance, Rab5 is involved in early endocytosis^23,26^, Rab7 is involved in the late endocytic pathway and protein degradation^27^ and Rab11 regulates protein recycling^28^. In particular Rab-GTPase family members have been shown to regulate membrane expression level of ion channels^7,29–32^.

Here we show that inhibition of Rab-GTPase is statin-specific. We show that endosomal localization of Rab5 was inhibited in a statin-specific manner, with stronger effects observed for fluvastatin, followed by simvastatin, atorvastatin and with rosuvastatin showing only a limited effect. Our data show statins have similar specificity on inhibition of other Rab-GTPases. To investigate statin-specificity on downstream targets, we investigated statin effect on Rab5-cPKC-mediated IKs channel internalization. Our data show statin-specific effects on IKs channel internalization both in heterologous system and cardiomyocytes. Our results indicate a novel statin-specific effect that may allow tailoring of specific statins for patients with disease-associated high cPKC activation. Most importantly, this is the first time that molecular mechanism for non-cholesterol-reducing and statin-specific effects are uncovered.

## Methods and Materials

### Constructs and chemicals

The following plasmid constructs were used in current study: hKCNQ1-GFP, hKCNE1 (donated by Dr. Robert Kass); HA-α_1A_ adrenergic receptor^33^; Rab5-GFP, Rab7-GFP and Rab11-GFP (donated by Dr. Gregory Tall); eGFP^33^. GFP-tagged protein kinase C isozymes, PKCα-GFP, PKCβI-GFP, PKCβII-GFP and PKCε-GFP were kindly provided by Dr. Naoaki Sato (Kobe University, Kobe, Japan) and have been previously described in details^34^. Adenovirus KCNQ1-GFP (Genebank NM_000218) and adenovirus KCNE1 (Genebank BC046224) were designed and amplified by Vector Biolabs. Cell permeable TAT-conjugated cPKC activator (KAC1–1, SVEIWD Cys – Cys TAT_47–57_)^35^, and a control peptide containing the HIV-TAT sequence (C1, TAT_47–57_)^36^ were gifts from KAI Pharmaceuticals (South San Francisco, CA). The cell permeable pseudo-RACK1 activator peptide (KKWKMRRNQFWIKIQRC-CSVEIWD, containing a disulfide bridge between 17 and 1, Tocris Bioscience) was diluted in water and applied to the extracellular media at 1 μM concentration. Statins and geranylgeranyl pyrophosphate (GGPP, ammonium salt) were purchased from Cayman Chemical Company. All other chemicals were purchased from Sigma Aldrich. Peptides (1 µM) and phenylephrine (30 µM) were water soluble and applied directly to either the extracellular recording solution (see below) or culture media for overnight treatment, as indicated. The stock solutions of statins were made in DMSO (10000×).

### Cell culture and transient expression

HEK293T cells (ATCC: The Global Bioresource Center) were cultured in 35-mm petri dishes in DMEM (Corning Cellgro, Cat# 15–013-CV) supplemented with 10% FBS (Sigma-Aldrich) and 1% GlutaMax (Cellutron Life Technologies) under 5% CO_2_ at 37 °C. Cells were transiently transfected with the plasmid DNA of interest using FuGene HD following manufacturer’s instruction (Promega). Equal amount of plasmid DNA of KCNQ1-GFP and KCNE1 was used (1.5 μg, 1:1 ratio). For Rab-GFPs, 0.3–0.5 μg plasmids of Rab5-GFP, Rab7-GFP and Rab11-GFP each were transfected. For PKC isozymes translocation experiments, 0.5 μg plasmid of PKC isozymes and 3 μg plasmid of HA-α1A-AR were co-transfected. Confocal images were conducted 48–56 hours after transient expression.

### Neonatal cardiomyocyte isolation

All animal care followed UCAR guidelines. Neonatal mouse ventricular cardiomyocytes were isolated from P0 neonates. The ventricles were minced in DMEM and digested in 4 changes of 0.8 mg/mL collagenase II (305U/µg, Worthington Biochemicals) at 37 °C in a small glass vial with a mini stir bar. Cells from each digestion were pooled and collected on ice, filtered through a 70 μm filter, and pelleted. Non-adherent neonatal ventricular cardiomyocytes were separated from the adherent fibroblasts by differential plating and plated on laminin (Sigma-Aldrich) coated glass bottom dishes.

### Adult rat cardiomyocyte isolation and infection

All animal care followed UCAR guidelines. Ventricular cardiomyocytes from adult female rat hearts were isolated as previously described^37^. Briefly, heart was extracted, placed in ice-cold Control Solution (in mM: 133.5 NaCl, 4 KCl, 1.2 NaH_2_PO_4_, 10 HEPES, 1.2 MgSO_4_, 11 Glucose. pH value was adjusted to 7.4 with NaOH), cannulated via the aorta, and placed on the Langendorff perfusion system. All solutions were oxygenated (95% O_2_) during perfusion and kept at 37 °C. The hearts were initially perfused with calcium containing solution for 5 min followed by calcium free Control Solution with added 0.1% BSA (Control Solution-BSA) for 5 min and collagenase containing solution (0.09% w/v) for 20 to 30 minutes or until heart was soft to touch. The ventricles were than isolated and minced. Cardiomyocytes were dissociated by mechanical trituration with glass Pasteur pipettes of different tip sizes. Cardiomyocytes were collected by gravity. Calcium was progressively increased using Control Solution-BSA with added calcium (µM: 50, 100, 200, 500 and 1000 CaCl_2_)_._ Cardiomyocytes were plated in glass bottom dishes pre-coated with laminin for 2 h, and incubated at 37 °C, 5% CO_2._ After 2 h, cardiomyocytes were infected with adenovirus at a ratio of 1 Av-KCNQ1-GFP: 1 Av-KCNE1 (1 × 10^8^ p.f.u./ml), overnight in 1 ml of culture media (Medium 199, Corning Cellgro, Cat# 10–060-CV) supplemented with 1–2% penicillin/streptomycin. Cardiomyocytes were washed with fresh media daily. Confocal images were taken 40–48 hours after infection.

### Cell treatment

For all overnight treatments cells were pre-treated at 37 °C with chemicals directly added to the growth medium. Following overnight treatment, cells were treated for 90 min with either peptides or Phe (supplemented with overnight treated chemical) added to the extracellular recording solution (in mM: 145 NaCl, 5.4 KCl, 1.8 CaCl_2_, 1 MgCl_2_, 10 HEPES, 10 D-Glucose, pH adjusted to 7.4 with NaOH)^7^ at 37 °C. Confocal images were taken at room temperature immediately after treatment.

### Confocal microscopy imaging

HEK293T cells were transfected with the plasmids of interest, specified in each experiment. 6 h after transfection, cells were split on glass bottom dishes (MatTek Corporation). Forty-eight hours after transfection, cells were washed two times with PBS without calcium or magnesium (Quality Biological) and then incubated at 37 °C for treatment in the extracellular recording solution (see above in Cell Treatment). After treatment, fluorescent images of the cells were taken with a confocal microscope (FV1000 Olympus, lenses: 60x oil). Confocal images were analyzed with ImageJ software to obtain KCNQ1-GFP fluorescence ratio between membrane and cytosolic regions with background subtracted for each cell (membrane/cytosol expression ratio: M/C ratio)^34,38^, as well as PKC isozyme fluorescence ratio. Rabs fluorescence ratio was measured between nucleous and cytoplasmic regions. Normalized membrane localization (Norm Memb) of KCNQ1-GFP was obtained by dividing the ratio of membrane/cytoplasm fluorescence above unit by the membrane to cytoplasm fluorescence above unit of the control condition at the day of the experiment ((M/C-1)/((M/C)_ctrl_−1)) (see details^7^). For adult cardiomyocyte confocal analysis, membrane/cytosol fluorescence of Av-KCNQ1-GFP was quantified in a rectangular area perpendicular to the cell membrane. Rectangular selection was done avoiding the T-tubules.

For PKC translocation experiments, HEK293T cells were co-transfected with 0.5 µg plasmid of PKC isozymes (PKCα-GFP, PKCβI-GFP, PKCβII-GFP or PKCε-GFP) and 3 µg plasmid of HA-α_1A_-AR. PKC isozymes were over-expressed for 48–72 hours before confocal images were taken. PKC isozymes localization was measured before and after acute stimulation of α1A adrenergic receptor with the agonist phenylephrine (30 µM) using a confocal microscope and the ratio of membrane to cytoplasm (M/C) fluorescence. PKC translocation was measured immediately after Phe stimulation for PKCα-GFP (<1 min) and 2–10 min after stimulation for the other PKC isozymes due to their kinetics of translocation^34^. For experiments on statin effects on PKC isozymes, cells were treated with 2 µM statins overnight. Statins were present in the solution during the acute stimulation experiment. To assure that α1A-AR was expressed, we only measured cells that show translocation upon phenylephrine stimulation. Only a small number of cells showed no translocation in all conditions tested (<10%).

### Immunochemistry

For immunocytochemistry on neonatal cardiomyocytes: after overnight treatments with 2 μM statins, neonatal cardiomyocytes were fixed with ice cold 4% paraformaldehyde solution in PBS (Santa Cruz Biotechnology) for 30 minutes at room temperature and subsequently washed 3 times with PBS. Cells were then permeabilized for 10 minutes with PBS supplemented with 0.25% triton X100 and washed 3 times with PBS. Subsequently, cells were slowly agitated in 5% BSA PBS solution for 40 minutes. Primary Rab5 antibody (C8B1, Rabbit mAb from Cell Signaling Technology) was applied (1/75 in PBS + 1% BSA) for 1.5 h at room temperature, followed by 3 times wash with PBS. Secondary antibody (Donkey anti-rabbit IgG HandL, Alexa Fluor 488) was applied for 1 h in 1% BSA PBS solution (1/1000), followed by 3 times wash with PBS. The nucleus staining solution (Hoechst 33258, Pentahydrate, Thermofisher Scientific) was applied directly to the cells for 10 minutes before imaging. For immunoblotting on HEK293T cells: after overnight 2 μM statin treatment, HEK293T cells were harvested with Laemmli buffer supplemented with phosphatase and protease inhibitor (Thermo Fisher Scientific). Samples were sonicated, centrifuged for 2 min at 15,000 g and supernatants were harvested as whole cell lysate. The samples were run on a 7% Acrylamide gel or with 4–20% Precast Protein Gels (Bio-Rad) under 95 V for 2 h. Antibodies against protein kinase C (PKC) (ab19031, Abcam) or GAPDH (sc-48166, Santa Cruz technology) were used and recognized by donkey anti-rabbit IgG antibodies (LI-COR Biosciences). The membranes were visualized using LI-COR (LI-COR Biosciences) and analyzed with ImageJ software.

### Cell viability

HEK293T cells were cultured in a 96-wells plate for 24 h at 37 °C, 5% CO2 in DMEM supplemented with 10% FBS and 1% GlutaMax and treated with 3 µM of statins overnight. Control conditions are vehicle (0.01% DMSO) and 8% ethanol treatment. Cells were then washed once with PBS and detached using Accutase (30 µl/well for 2–3 minutes). Cells were dissociated using a pipette, and digestion was stopped using DMEM supplemented with 0.1% Trypan blue solution (100 µl/well). Cell viability was measured using Life Sciences Countless II FL cell counter.

### Statistical analysis

All results are shown as mean ± standard error of the mean (SEM). For comparison of two groups a t-test was used. When the means of more than two independent groups were compared, One-way ANOVA, followed by Dunnet’s test was done. The significance level set at P < 0.05.

## Results

### Subcellular localization of Rab-GTPases is statin-specific

**Figure 1:**
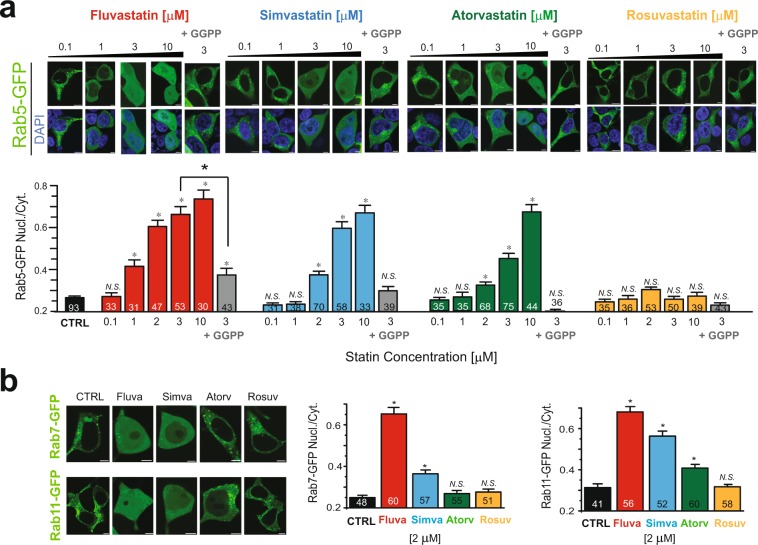
Statins specificity of Rab-GTPase inhibition. (**a**) *Top*, Typical confocal microscopy images of HEK293T cells over-expressing Rab5-GFP after overnight statin treatment at the indicated concentration in the presence or absence of 10 µM GGPP. *Bottom*, Summary of Rab5 nucleus/cytoplasm fluorescence ratio of the experiment performed in top panel. (**b**) *Left*, typical confocal images of HEK293T cells over-expressing either Rab7-GFP or Rab11-GFP in control conditions, or after 2 μM statin treatment for overnight. *Right*, Summary of Rab7 and Rab11 nucleus/cytoplasm ratio. Scale bars, 5 μm. *p < 0.05 (number of cells analyzed).

Statins have been shown to inhibit isoprenylation of Rab-GTPases and therefore inhibit protein localization and function^39,40^. Therefore, we investigated the dose dependent effect of statins on Rab5 subcellular localization. We treated cells expressing Rab5-GFP overnight with four different statins (fluvastatin, simvastatin, atorvastatin or rosuvastatin) at increasing doses (0.1 to 10 μM). In untreated cells (vehicle, 0.01% DMSO) the fluorescence-tagged Rab5-GFP constructs were present in intracellular compartments and largely absent in the cell nucleus (Fig. 1a, black bars for controls). After fluvastatin treatment, Rab5 showed a significant increase in diffuse localization (measured by nuclear to cytoplasmic fluorescence ratio) at concentration above 1 μM. Simvastatin and atorvastatin also showed a dose-dependent effect on Rab5 localization (Fig. 1a). However, rosuvastatin showed no effect, even at the highest concentration tested (Fig. 1a).

Prenylation occurs by covalent addition of two types of isoprenoids to cysteine residues at the carboxyl terminus of the proteins that possess prenylation motifs, farnesyl pyrophosphate (FPP) or geranylgeranyl pyrophosphate (GGPP). Statins inhibit protein prenylation by decreasing levels of both FPP and GGPP^41^. Prenylation of Rab-GTPases requires GGPP. Thus, to confirm the statin effect was mediated by Rab prenylation, we measured the effect of direct application of GGPP. GGPP completely restored Rab5 cytoplasmic localization in the presence of statins (simvastatin and atorvastatin, while partially restoring the strong effect of fluvastatin on Rab5 localization). GGPP had no significant effect on Rab5 localization in the presence of rosuvastatin. Our results suggest that inhibition of prenylation of Rab-GTPase is responsible for the statin effects (Fig. 1a). Consistent with an effect of statins in Rab prenylation, unprenylated Rabs were previously shown to have diffuse cellular and nuclear localization^20^.

We also investigated the statin specificity on other Rab-GTPase family members. We tested the effect of statins (2 µM) on Rab7 and Rab11. Fluvastatin and simvastatin treatment increased Rab7 diffuse localization (Fig. 1b). Treatment with all but rosuvastatin increased Rab11 diffuse localization (Fig. 1b). Similar to the effect on Rab5, on both Rab 7 and Rab 11 fluvastatin showed the strongest effect, followed by simvastatin and atorvastatin. On either Rab7 or Rab11 rosuvastatin showed no effect.

Overall, our results show a differential effect of statins on decreasing membrane targeting of Rabs, with the strongest effect observed with fluvastatin, followed by simvastatin, atorvastatin and with limited effect observed for rosuvastatin.

### Subcellular localization of endogenous Rab-GTPases is statin-specific in cardiomyocytes

**Figure 2:**
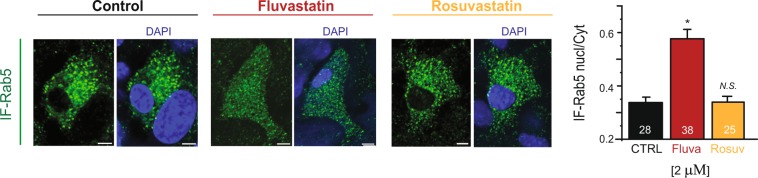
Statins specificity of Rab5-GTPase inhibition in neonatal cardiomyocytes of mice. *Left*, typical confocal images of the immunofluorescence of endogenous Rab5 labeled with anti-Rab5 antibodies in neonatal mice cardiomyocytes after overnight statin treatment as indicated. *Right*, summary of the experiments done to the left. Scale bars, 5 µm. *p < 0.05 (number of cells analyzed).

In order to confirm statin-specific regulation of Rab-GTPase localization in the native system, without Rab-GTPase overexpression, we used isolated neonatal ventricular cardiomyocytes from mice. After overnight treatments with 2 μM statins, neonatal cardiomyocytes were fixed and immunostained with Rab5 antibody. We focus on Rab5 because our recent work shows it underlies KCNQ1/KCNE1 channel internalization in both heterologous expression systems and cardiomyocytes^7^. We measured the effects of fluvastatin and rosuvastatin on endogenous Rab5 localization in neonatal cardiomyocytes. In untreated cells, Rab5 exhibited endosomal localization and was largely absent in the nucleus (Fig. 2). Consistent with our results in the heterologous system, fluvastatin treatment caused significant inhibition of endosomal Rab5 localization and cardiomyocytes exhibited a diffuse cellular localization (as measured by nuclear/cytoplasmic ratio) while rosuvastatin treatment did not have any significant effects on Rab5 localization (Fig. 2).

### Statin inhibits Rab5-cPKC-mediated effects on KCNQ1/KCNE1 channels in a statin-specific manner

**Figure 3:**
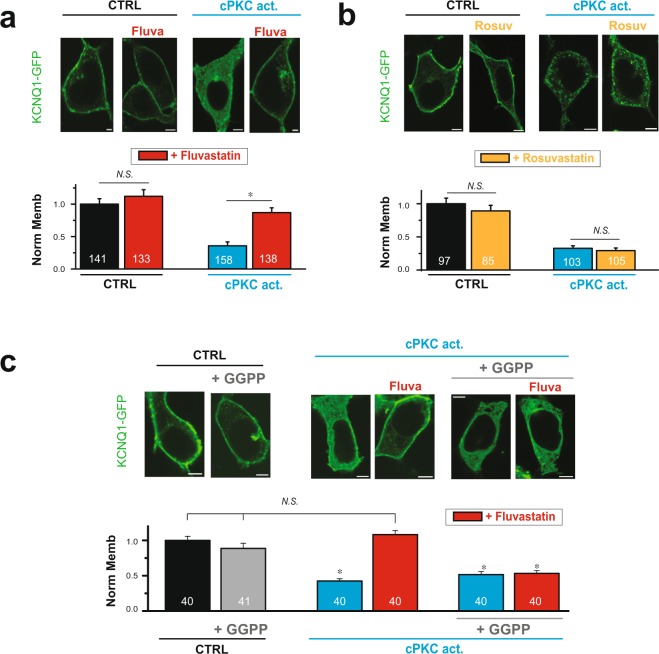
Fluvastatin, but not rosuvastatin restores membrane localization of KCNQ1/KCNE1 channels in response to sustained cPKC activation. HEK293T cells were transiently expressing KCNQ1-GFP and KCNE1. (**a**) *Top*, Typical confocal images of cells treated with 1 µM either control or cPKC activator peptide for 90 min in the presence or absence of 1 µM fluvastatin overnight pre-treatment. *Bottom*, Summary of KCNQ1-GFP membrane localization in cells treated as indicated. (**b**) *Top*, Typical confocal images of cells in the presence and absence of overnight rosuvastatin treatment (1 µM) followed by 90 min treatment with either control or cPKC activator peptide as indicated. *Bottom*, Summary data of KCNQ1-GFP membrane localization in cells treated as indicated. (**c**) *Top*, Typical confocal images of cells treated for 90 min with cPKC activator peptide in presence of either 1 µM fluvastatin or 10 µM GGPP and 1 µM fluvastatin (overnight pre-treatment for both conditions). *Bottom*, Summary of KCNQ1-GFP membrane localization in cells treated as indicated. control = control peptide C1 (panels a and b), vehicle (panel c); cPKC act. = cPKC activator peptide KAC1–1 (panels a and b) and pseudoRACK1 (panel c). Scale bars, 5 µm. *p < 0.05 (number of cells analyzed).

To date, at least 12 PKC isozymes have been identified in mammalian tissues and are ubiquitous within the cell. Members of one subfamily require Ca^2+^ for activation and include PKCα, βI, βII, and γ (cPKC). Members of the second subfamily are Ca^2+^-independent, and include PKCδ, ε, η, and θ. cPKCs in particular are strongly up-regulated and chronically activated in disease^2,3,42–50^. Members of the cPKC family (PKCα, βI, βII) and PKCε have been shown to be expressed in the heart and translocate to the membrane in response to G-protein receptor stimulus^51–54^. Our recent work showed that prolonged cPKC stimulus (90 min, 37 °C) promoted KCNQ1/KCNE1 channel internalization and consequent decrease in channel function, which is mediated by Rab5 and inhibited by fluvastatin^7^. Our work showed that the channel was internalized via-Rab5 endosomes and target for recycling in Rab11 endosomes, while Rab7-mediated degradation was not involved, even with prolonged (overnight) stimulation. In order to investigate whether statin-specificity of Rab-GTPase had functional consequences for KCNQ1/KCNE1 channel membrane expression, we compared the effects of the Rab-GTPase targeting fluvastatin to the Rab-GTPase non-targeting rosuvastatin on channel membrane localization in response to cPKC activation. KCNQ1-GFP together with the auxiliary subunit KCNE1 was expressed in HEK293T cells. Cells were treated with statins overnight, and subsequently stimulated with a cell permeable activator peptide specific for cPKC^7,35^. For statin-untreated cells, cPKC activation caused a strong decrease in channel membrane localization. In fluvastatin-treated cells, the effect of cPKC activation was inhibited (Fig. 3a). We show that direct GGPP applications had no significant effect on channel localization and cPKC inhibition, but abolished the fluvastatin effect, consistent with our recently published data^7^ (Fig. 3c). Rosuvastatin treatment, in contrast to fluvastatin did not affect cPKC-mediated internalization of KCNQ1 (Fig. 3b).

Our results indicate that inhibition of Rab5-mediated channel internalization is statin-specific, consistent with the effects of statin-specific effect on Rab-GTPases.

### Statin inhibition of Rab5-cPKC-mediated effects on KCNQ1/KCNE1 channels is not due to effect on cell viability or PKC expression or function

**Figure 4:**
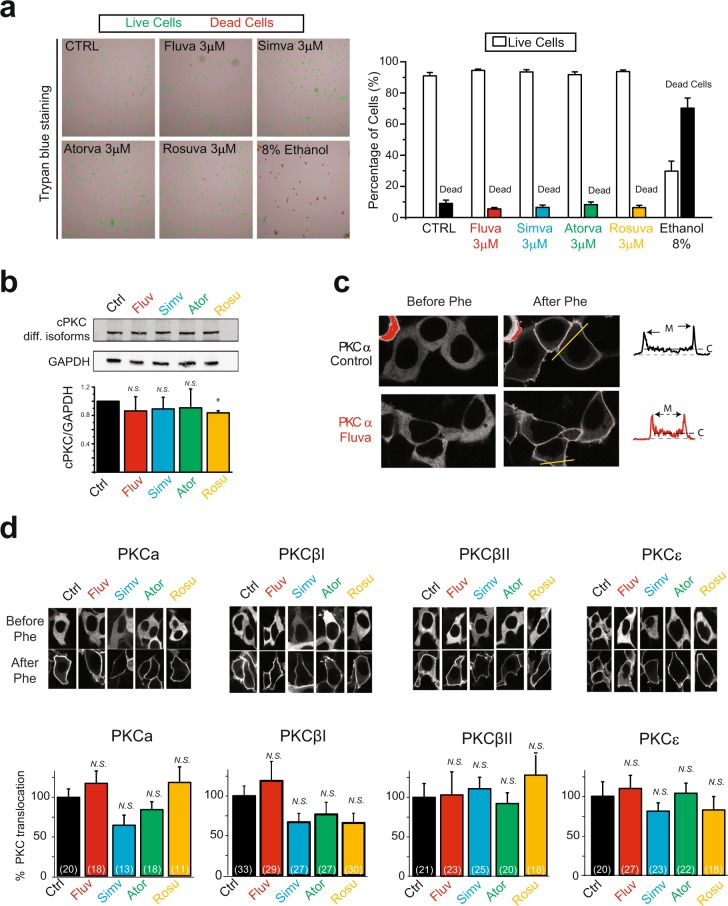
Statins did not change cell viability, or expression of PKC isozymes or their functions. (**a**) Statin treatment did not change cell viability. *Left*, representative images of HEK 293 T cells treated overnight with 3 μM of statins as indicated. Cells were labeled after staining with Trypan blue for viability count. Control cells were treated with either 0.01% DMSO (CTRL) or 8% ethanol. *Right*, summary data of experiments performed to the left. (**b**) Statin treatment did not change cPKC expression level. Typical immuno-blot and summary data of endogenous cPKC expression in HEK293T cells after 2 µM statin treatment for overnight. cPKC isozymes are recognized by an anti-cPKC antibody. *p = 0.05 (**c**) Typical confocal images of HEK293T cells expressing GFP tagged PKCα before and after acute phenylephrine treatment (within 1 minute) for stimulation of overexpressing α1A-AR (Phe, 30 µM), in the presence and absence of overnight fluvastatin treatment (2 µM) as indicated. Fluorescence profile was measured at the cell cross section indicated by the yellow line and shown in the inset to the right. M and C indicate plasma membrane and cytoplasm respectively. (**d**) *Top*, typical confocal images of PKCα-GFP, PKCβI-GFP, PKCβII-GFP and PKCε-GFP before and after acute Phe treatment (30 μM, details in Methods) at the absence and presence of overnight statins treatment (all at 2 μM). *Bottom*, summary of the translocation of PKC isozymes in experiments conducted as above. *p < 0.05 (number of cells analyzed).

Statins have been suggested to have direct effects on PKC, increasing kinase activity and expression^55–57^ as well as decreasing cell viability^58^. To confirm the statin effects we observed on Rab5-cPKC-mediated internalization of KCNQ1/KCNE1 channels are not mediated by direct effects on viability or direct PKC expression or activation, we first measured cell viability using Trypan blue staining and a cell counter. We treated cells overnight with fluvastatin, simvastatin, atorvastatin or rosuvastatin (3 μM). For all statin treatment, statins did not significantly affect cell viability at concentrations used in our experiment. As a positive control we showed that ethanol treatment strongly decreased cell viability (Fig. 4a).

We next investigated whether statins directly affected PKC. We investigated whether a direct statin-specific effect on PKC may underlie the statin-specific inhibition of cPKC-mediated channel internalization. First, we measured protein expression levels of endogenous cPKC isozymes after treatment with fluvastatin, simvastatin, atorvastatin and rosuvastatin (2 µM, overnight). In contrast to published data for higher PKC activity that suggest statins activate PKC^56^, in our experiments, for all treatments, a trend was observed for statins to mildly inhibit cPKC isozyme expression. No significant effect was observed for fluvastatin, simvastatin and atorvastatin, while rosuvastatin inhibited approximately 15% of cPKC expression (Fig. 4b). No significant differences were observed among the different statins.

Second, we tested whether statins differentially affect PKC function. For that we measured PKC translocation to the plasma membrane in response to agonist stimulation. We expressed GFP-tagged cPKCs (PKCα, PKCβI, PKCβII) and the calcium independent PKCε in cells together with α1A adrenergic receptor (α1A–AR). We measured localization of PKC before and after stimulation with an α1A-AR agonist, phenylephrine (Phe, 30 μM). Before stimulation, all isozymes were localized in the cytoplasm, with or without statin treatment (Fig. 4c-d). After agonist stimulation, cells treated with statins showed no difference in PKC translocation compared to control cells (Fig. 4c). This finding indicated that statin treatment did not significantly affect agonist-mediated translocation of any PKC isozyme (Fig. 4c-d).

Overall, our results suggest that the statins effects observed are statin-specific. In addition our data indicate that statin-specific inhibition of PKC-mediated channel internalization is not a result of direct statin effects on PKC and cell viability.

### Statin inhibits Rab5-cPKC-mediated effects on KCNQ1/KCNE1 channels in a statin-specific manner in adult cardiomyocytes

**Figure 5:**
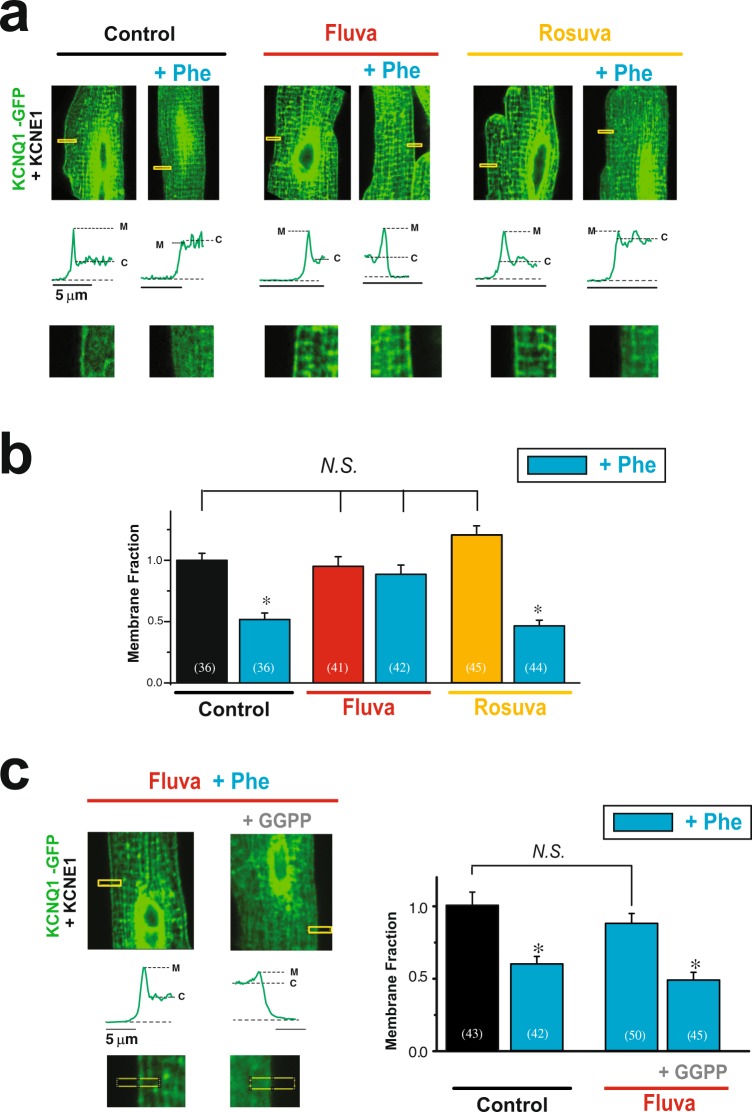
Fluvastatin inhibits cPKC-induced internalization of KCNQ1/KCNE1 channels in adult rat cardiomyocytes. Adult rat cardiomyoctyes were infected with Av-KCNQ1-GFP and Av-KCNE1. (**a**) Typical confocal images of isolated rat ventricular myocytes expressing KCNQ1-GFP and KCNE1 treated with agonist phenylephrine (Phe, 30 μM) for 90 min to stimulate the endogenous α1-AR in the presence and absence of overnight statin treatment (fluvastatin and rosuvastatin, both at 1 μM) as indicated. Insets show boxed areas in magnification and the fluorescence profiles of KCNQ1-GFP. M and C indicate sarcolemma membrane and cytoplasmic fluorescence. (**b**) Summary data of normalized membrane localization of KCNQ1-GFP from experiments described in panel (a). (**c**) *Top*, Typical confocal images of cells treated for 90 min with agonist phenylephrine (Phe, 30 µM) in presence of either 1 µM fluvastatin with or without 5 µM GGPP (overnight pre-treatment for both conditions) as indicated. *Bottom*, Summary of KCNQ1-GFP membrane localization in cells treated as indicated. Scale bars, 5 μm, *p < 0.05, (number of cells analyzed).

In order to confirm statin-specific regulation of Rab5-cPKC mediated internalization of the channel in the native system, we investigated the effect of prolonged stimulation of Gq-coupled receptors (GqPCR) on channel membrane localization in isolated rat ventricular myocytes. Adult rat cardiomyocytes do not express functional endogenous KCNQ1/KCNE1 channels, allowing study of the human channel^59^. cPKCs are activated in response to activation of G protein-coupled receptor of the Gq family and have been shown to be activated by stimulation of α adrenergic receptor in cardimyocytes^33,34,60–63^. We stimulated cardiomyocytes expressing Av-KCNQ1-GFP and Av-KCNE1 with the α1A-AR agonist (Phe, 30 µM, 37 °C) overnight. Sarcolemma membrane localization of KCNQ1-GFP was measured using confocal microscopy images. As in HEK293T cells, before stimulation, KCNQ1-GFP (in green) is strongly localized at the sarcolemma membrane (Fig. 5). Sarcolemma membrane expression of KCNQ1-GFP was strongly inhibited after cell stimulation with Phe (Fig. 5). Changes in KCNQ1-GFP membrane localization in response to agonist stimulation were inhibited by overnight treatment with fluvastatin but were unaffected by rosuvastatin treatment (1 µM) (Fig. 5), suggesting the statin-specificity is conserved in the native environment. To confirm the Rab-GTPase target of fluvastatin in the native system, we showed that GGPP treatement abolished the fluvastatin effect (Fig. 5c). Our results indicate that statin specifically inhibits Rab5-GTPase-mediated internalization of the channel in cardiomyocytes.

## Discussion

Our data show that statins inhibit Rab-GTPase localization in a statin-specific manner, with the stronger effects observed for fluvastatin and simvastatin and with no effect observed for rosuvastatin even at high concentrations. We show that this specificity is conserved in cardiomyocytes. In addition, we show that statin inhibition of Rab5-cPKC-mediated regulation of ion channel membrane expression was statin-specific both in heterologous systems and cardiomyocytes, suggesting that statin-specific effects on Rab-GTPases allow differential regulation of Rab-targeted proteins. cPKC is known to be activated in a number of disease states^43,63–68^. Our data suggest that patients whose diseases are associated with increase in cPKC activity may further benefit from hydrophobic statins such as fluvastatin, simvastatin and atorvastatin. This effect may allow better tailoring of the statin treatment to patients’ disease phenotypes.

Statins block cholesterol synthesis and have been shown to have a number of health benefits^69–71^. Statins are also known to prevent isoprenylation of Rabs and inhibit their function as trafficking regulators^39,40,72^. For example, Procino and collegues have shown that aquaporin AQP2 is accumulated at the plasma membrane after inhibition of Rabs by statins^73^. Statins can be differentiated as either hydrophilic or lipophilic based on their water solubility. Among the four statins tested, rosuvastatin, a hydrophilic statin, was the only statin that showed no significant effect on Rab5 even at the highest concentration and consequently did not affect channel internalization, suggesting that hydrophobicity may underlie the statin specificity observed.

KCNQ1 and KCNE1 subunits form the cardiac IKs current, one of the major channels responsible for cardiac action potential repolarization^22^. Mutations in KCNQ1 that lead to decrease in IKs currents are the most common form of inherited cardiac arrhythmia^74^. Our recent work suggest that KCNQ1/KCNE1 internalization in response to stress stimulus may underlie QTc prolongation and increased arrythmogenesis in heart disease and diabetes^7^. Here we show that hydrophobic statins (fluvastatin, simvastatin and atorvastatin), but not rosuvastatin, inhibit KCNQ1/KCNE1 internalization in response to stress stimulus. Thus, our results suggest hydrophobic statins, by targeting Rab-GTPases, may have an antiarrhythmic effect by preventing decrease in IKs currents and pathological QT prolongation. Although statins have a number of effects that are not specific to KCNQ1/KCNE1 channel internalization, their wide spread use suggests that they may safely be used. Indeed, statins have been shown to reduce the rate of ventricular fibrillation in heart disease patients^2,3^, although the mechanism underlying these effects was not elucidated. More recently, in smaller studies, statin therapy was shown to shorten QTc and QTc dispersion in heart failure patients^16^ and suppress superventricular arrhythmias^17,18^.

Serum concentration of fluvastatin in healthy subjects is on the [0.1–1.0 µM] range^75^, thus the effects we observe are within pharmacological levels of the drug. In addition, plasma concentrations of simvastatin and lovastatin have been shown to increase up to 20-fold in the presence of cytochrome P450 inhibitory drugs^8,76^. Thus, although higher concentrations of simvastatin and atorvastatin are required for the effects observed, concentrations used are relevant pharmacologically.

Despite hydrophilic statins such as rosuvastatin showing strong efficacy in lowering cholesterol and preventing cardiovascular disease^77,78^, a number of studies have suggested increased benefits of lipophilic statins^20,21,79^. For instance the PRIMO study (Prédiction du Risque Musculaire en Observationnel; Prediction of Muscular Risk in Observational conditions) investigated muscular symptoms in a population of approximately 8,000 hyperlipidemic patients treated with statins^80^. This study has concluded that fluvastatin has less muscle related symptoms compared to atorvastatin and simvastatin. Based on the partition coefficient from water to n-octanol, the lipophilicity of the statins used are: simvastatin 1.5–1.75, fluvastatin 1.0–1.25, atorvastatin 1.2–1.25 and rosuvastatin −0.25-(−0.5)^20^. Based on statin lipophilic properties, rosuvastatin and pravastatin are commonly clinically referred as hydrophilic, while all other statins, including fluvastatin, simvastatin and atorvastatin, are referred as hydrophobic or lipophilic^20^. Our work pointed to lipophilic statins, in particular fluvastatin, having a stronger effect on Rab-GTPases, thus protective Rab-GTPase-mediated effects may be related to the lower muscular symptoms observed by fluvastatin.

In conclusion we uncovered that statin regulation of Rab-GTPase is statin-specific with the strongest effects observed for fluvastatin. We showed that fluvastatin, in contrast to rosuvastatin, can have a beneficial effect in cardiac electrophysiology by restoring IKs channel membrane expression via Rab-GTPase targeting. Thus, specific statin treatment may be tailored to patients with specific IKs dysfunction and arrhythmic risk. Because Rabs are responsible for control of trafficking of a number of membrane proteins, statin-specific Rab inhibitory effects may be important in many pleiotropic effects of statins.

## Data Availability

Materials, data and associated protocols promptly available upon request.
